# A surrogate FRAX model for the Kyrgyz Republic

**DOI:** 10.1007/s11657-020-00743-2

**Published:** 2020-05-06

**Authors:** O. Lesnyak, A. Zakroyeva, O. Lobanchenko, H. Johansson, E. Liu, M. Lorentzon, N. C. Harvey, E. McCloskey, J. A. Kanis

**Affiliations:** 1 Mechnikov North West State Medical University, St. Petersburg, Russia; 2 Ural State Medical University, Yekaterinburg, Russia; 3 I.K. Akhunbaev Kyrgyz State Medical Academy, Bishkek, Kyrgyz Republic; 4 Mary McKillop Institute for Health Research, Australian Catholic University, Melbourne, Australia; 5 Geriatric Medicine, Institute of Medicine, Sahlgrenska Academy, University of Gothenburg, Gothenburg, Sweden; 6 MRC Lifecourse Epidemiology Unit, University of Southampton, Southampton, UK; 7 Centre for Metabolic Bone Diseases, University of Sheffield, Sheffield, UK

**Keywords:** FRAX, Fracture probability, Epidemiology, Hip fracture, Kazakhstan, Surrogate, Kyrgyz Republic

## Abstract

***Summary*:**

The hip fracture rates from Kazakhstan were used to create a surrogate FRAX® model for the Kyrgyz Republic.

**Introduction:**

The International Society for Clinical Densitometry and International Osteoporosis Foundation recommend utilizing a surrogate FRAX model, based on the country-specific risk of death, and fracture data based on a country where fracture rates are considered to be representative of the index country.

**Objective:**

This paper describes a surrogate FRAX model for the Kyrgyz Republic.

**Methods:**

The FRAX model used the incidence of hip fracture from the neighbouring country of Kazakhstan and the death risk for the Kyrgyz Republic.

**Results:**

Compared with the model for Kazakhstan, the surrogate model gave somewhat higher 10-year fracture probabilities for men between 60 and 80 years of age and lower probabilities for men above the age of 80. For women the probabilities were similar up to the age of 75–80 years and then lower. There were very close correlations in fracture probabilities between the surrogate and authentic models (1.00) so that the use of the Kyrgyz model had little impact on the rank order of risk. It was estimated that 2752 hip fractures arose in 2015 in individuals over the age of 50 years in the Kyrgyz Republic, with a predicted increase by 207% to 8435 in 2050.

**Conclusion:**

The surrogate FRAX model for the Kyrgyz Republic provides the opportunity to determine fracture probability among the Kyrgyz population and help guide decisions about treatment.

## Introduction

In 2008, the WHO Collaborating Centre for Metabolic Bone Diseases at the University of Sheffield, UK, developed algorithms to compute age-specific fracture probabilities in women and men from readily obtained clinical risk factors (CRFs) and BMD measurements at the femoral neck (http://www.shef.ac.uk/FRAX). The algorithm (FRAX®) was based on a series of meta-analyses using the primary data from population-based cohorts that identified several CRFs for fracture [1, 2]. FRAX models compute the probability of major osteoporotic fracture (hip, spine, distal forearm or proximal humerus) or hip fracture derived from the risk of fracture and the competing risk of death, both of which vary from country to country [3]. At present, FRAX models are available for 64 countries.

The availability of FRAX has stimulated studies that can be used for the generation of new FRAX models. Specific examples include Brazil, Mexico and Turkey [4]. The present study is a component part of the multicentre multinational population-based study in Eurasian countries (EVA study or ЭВА, in Russian). The broad aim of the study was to provide epidemiological information on fracture risk so that FRAX models could be created for Russia [5], Armenia [6], Belarus [7], Moldova [8], Kazakhstan [9], Uzbekistan and the Kyrgyz Republic. Ideally, data for both fracture incidence and death should be available for the construction of country-specific FRAX models. Unfortunately, the systems available to study the epidemiology of fracture proved to be inadequate in the Kyrgyz Republic.

Recognizing that data on hip and other fractures are not always available, the International Society for Clinical Densitometry and International Osteoporosis Foundation recommend using a surrogate FRAX model, based on the country-specific risk of death, and fracture data based on a country where fracture rates are considered to be representative of the index country [10]. Of the 66 countries for which a FRAX model is available, five FRAX country-specific models currently use surrogate data on fracture risk (Georgia, India, Palestine, Sri Lanka and Syria). The present report describes the development of a surrogate FRAX model for the Kyrgyz Republic.

## Methods

The Kyrgyz Republic is a landlocked country with mountainous terrain. It is bordered by Kazakhstan to the north, Uzbekistan and Tajikistan to the southwest and China to the southeast. The Kyrgyz Republic has an area of 191,800 square kilometres (74,054 square miles) with a population estimated at 6,460,458 in 2019 [11]. The population of the Kyrgyz Republic is young with a median age of 25.3 years. Only 16.3% of the population has an age of 65 years or more.

Kazakhstan was chosen as a surrogate country for the Kyrgyz Republic FRAX model since the epidemiology of hip fracture in Kazakhstan was derived from the Almaty region, close to the Kyrgyzstan border. Details of the Kazakhstan FRAX model are available elsewhere [9]. Kazakhstan belongs to the moderate-risk countries for hip fracture for men and women [3]. National mortality rates for Kyrgyzstan used data from the World Health Organization for 2014 [12].

For the purpose of comparing the authentic FRAX model for Kazakhstan and the surrogate model for the Kyrgyz Republic, the probabilities of a major osteoporotic fracture (hip, clinical spine, forearm and humeral fractures) and of hip fracture alone were computed in men and women at ages 50, 60, 70 and 80 years for all possible combinations of clinical risk factors at BMD T-scores between 0 and − 3.5 SD in 0.5 SD steps with a BMI set to 26 kg/m^2^ [13]. Thus, we considered all combinations of six risk factors and eight values of BMD giving a total of 512 combinations. Note that this was not a population simulation, but an array of all possible combinations. The correlation between the probabilities derived from the surrogate and authentic models was examined by piecewise linear regression with knots at the probabilities of 40% for the Kazakhstan probabilities of a major osteoporotic fracture and hip fracture. Tabular data were used to compare probabilities between the two versions at the 50th (median) percentile of the distribution of the Kazakh model. Differences in the Kyrgyz model from the Kazakh model at these percentiles were expressed as 95% tolerance intervals (TI).

The age- and sex-specific incidence in 2015–2017 was applied to the Kyrgyz population in 2015 to estimate the number of hip fractures nationwide. Additionally, future projections were estimated up to 2050 assuming that the age- and sex-specific incidence remained stable. Population demography was taken from the United Nations using the medium variant for fertility [14].

## Results

**Fig. 1:**
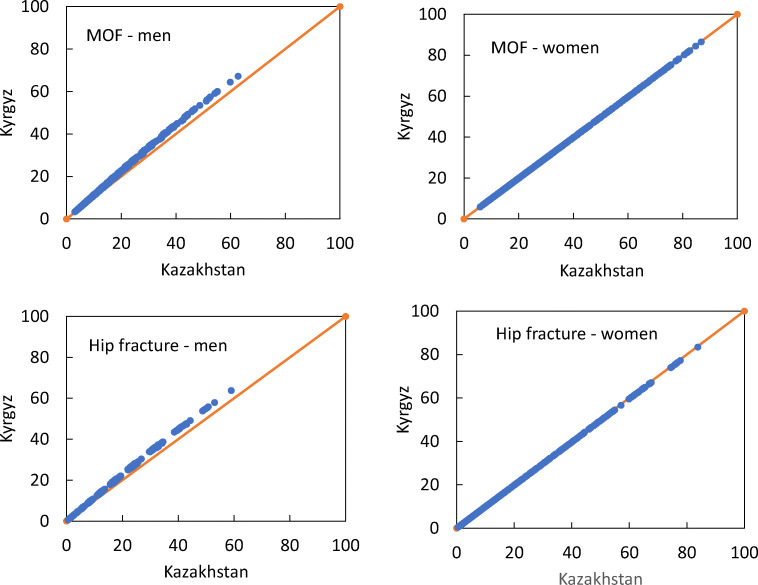
Comparison of 10-year probability of fracture using the surrogate FRAX tool for the Kyrgyz population and the Kazakh FRAX tool for multiple clinical scenarios at the age of 70 years. The left-hand panels show the comparison in men. The top panels relate to major osteoporotic fracture (MOF) and the lower panels to hip fracture probability. The diagonal line shows the line of identity

There was a close correlation between the FRAX model for Kazakhstan and the surrogate Kyrgyz model. In the case of women, the correlation coefficient was 1.0 with a slope of unity at all ages except for the age of 80 years. For men, the correlation coefficients were also high (*r* = 1.00), but the surrogate model gave somewhat higher probabilities than the Kazakh model at the age of 70 years. The relationship between the probabilities of a major osteoporotic fracture and hip fracture derived from the two versions of FRAX is shown for men and women age 70 years in Fig. 1. For men at the age of 70 years, the median value of the surrogate version was higher by about 12% for the probability of hip fracture and major osteoporotic fracture. At other ages, the difference was less than 3% (Table 1). For women the difference was less than 1% for ages below 80. For 80 years of age, the difference was 7%. Similarly, small and non-significant differences in fracture probabilities were noted at the 25th and 75th percentile of the probability distribution (data not shown).

**Table 1:** Probability (%) of a major osteoporotic fracture (MOF) or a hip fracture (with 95% tolerance intervals; TI) in men and women at the median of the probability distribution (Kazakh version) by age

	Men				Women			
	Kazakhstan	Kyrgyz Republic			Kazakhstan	Kyrgyz Republic		
Age	Median	Median	95% TI	*r* value	Median	Median	95% TI	*r* value
MOF								
50	22.8	22.9	22.8–22.9	1.000	24.1	24.1	24.1–24.1	1.000
60	19.6	20.2	20.0–20.4	1.000	25.6	25.6	25.5–25.6	1.000
70	15.5	17.4	16.9–17.9	1.000	27.3	27.1	27.1–27.2	1.000
80	17.6	17.3	17.0–17.6	1.000	28.9	27.1	26.7–27.5	1.000
Hip fracture								
50	6.9	6.9	6.9–7.0	1.000	4.8	4.8	4.8–4.8	1.000
60	6.8	7.0	6.9–7.2	1.000	6.0	6.0	6.0–6.0	1.000
70	8.3	9.4	9.0–9.8	1.000	10.7	10.6	10.6–10.7	1.000
80	12.2	11.9	11.6–12.2	1.000	16.8	15.7	15.4–16.0	1.000

### Fracture projections

Assuming that the fracture rates in Kazakhstan were representative for the Kyrgyz Republic, and based on the UN estimates of the Kyrgyz population for 2015, we estimated that the annual number of hip fractures in men and women age 50 years or older in the Kyrgyz Republic in 2015 was 2752, comprising 984 in men and 1768 fractures in women. The number of major osteoporotic fractures was 9851 comprising 3756 in men and 6095 in women. The number of hip fractures is expected to increase progressively by calendar year with an increase of 207% by 2050 (Table 2). The increase in hip fracture numbers is particularly great in women (226% in women and 172% in men) due to the longer life expectancy in women.

**Table 2:** Estimated total number of hip fractures (ICD-10 codes S72.0, S72.1, S72.2) in men and in women age 50 years and older in 2015 projected up to 2050 in the Kyrgyz Republic

	2015	2020	2030	2040	2050
Men	984	1148	1496	2042	2672
Women	1768	1983	2795	4315	5763
Total	2752	3131	4291	6357	8435
Increase (%)	–	14	56	131	207

## Discussion

The incidence of hip fracture in Kazakhstan was used to populate a surrogate FRAX model for the Kyrgyz Republic to compute the 10-year probabilities of hip and major osteoporotic fracture. In brief, the surrogate provided slightly higher estimates of fracture probability in men and similar or slightly lower estimates in women compared with the Kazakh model. The modest differences reflect the similarity in mortality between the two countries. Importantly, the differences had little impact on the categorization of risk, since there was little or no change in the rank order of fracture probability. In the clinical scenarios studied in this paper, the correlation coefficients between surrogate and Kazakh versions for fracture probability were 1.00, so that the one can be accurately predicted from the other. In other words, an individual at the 90th percentile of risk would still be at the 90th percentile of risk using the surrogate FRAX tool. Thus, any consequences of improving accuracy would reside in the absolute number generated and not in the rank order of risk. This is of little consequence to the management of patients or the interpretation of clinical studies. There is a useful analogy with the different DXA devices available, where a substantial difference in femoral neck BMD is seen between Hologic and Lunar machines, but the T-score derived from these is more or less identical [15]. However, marked difficulties arise when fracture probabilities are used in health economic analysis to inform practice guidelines.

There are obvious limitations to this study in that the assumption is that the fracture rates in the Kyrgyz Republic are similar to those in Kazakhstan. In addition to geographic proximity, peoples of Kazakhstan and Kyrgyzstan share several similarities. Kazakh and Kyrgyz nations are very close genetically [16]. Both countries have similar cultural and religious characteristics (prevailing religion, traditions, common Turkic language root) [17]. There is also commonality between the Almaty Region of Kazakhstan and Kyrgyzstan in major ethnic groups [18]. These observations do not test directly the similarity of fracture rates. Indeed, there are precedents for doubting the assumption, for example, the historical use of Romanian fracture rates to populate a surrogate Armenian model [6].

A minority of countries that have a FRAX model also have robust information on the risk of other major osteoporotic fractures. In the absence of such information, FRAX models are based on the assumption that the age- and sex-specific pattern of these fractures is similar to that observed in Malmo [19]. This assumption, used in the case of Kazakhstan, has been shown to be safe in studies reported from Canada [20], Iceland [21], USA [22], UK [23], Australia [24] and Moldova [8], despite differences in incidence between these countries [3]. This commonality of pattern is supported by register studies, which indicate that in those regions where hip fracture rates are high, so too is the risk of forearm fracture and spine fractures (requiring hospital admission) [25, 26].

The widespread availability of FRAX has resulted in its adoption in many practice guidelines worldwide [27]. The fracture probability equivalent to a woman with a prior fracture has been used as an intervention threshold in more than 30 countries. If the same threshold were applied to the Kyrgyz Republic, then intervention would be recommended with a probability of a major fracture that varied between 5.3 and 15% depending on age. The impact of such thresholds or alternative thresholds will require further study.

In summary, a surrogate FRAX model has been created for the Kyrgyz Republic. The model can provide the opportunity to determine fracture probability among the Kyrgyz population and help guide decisions about treatment.
